# Effects of Charged Surface on Electrochemical Sensitivity to Protein Dimerization

**DOI:** 10.1021/acs.analchem.5c05281

**Published:** 2025-10-31

**Authors:** Hana Černocká, Veronika Kasalová, Eva Tihlaříková, Vilém Neděla, Roman Hrstka, Veronika Ostatná

**Affiliations:** † Institute of Biophysics, The Czech Academy of Sciences, v.v.i., Královopolská 135, 61200 Brno, Czech Republic; ‡ Institute of Scientific Instruments, The Czech Academy of Sciences, v.v.i., Královopolská 147, 61264 Brno, Czech Republic; § Masaryk Memorial Cancer Institute, Regional Centre for Applied Molecular Oncology, Žlutý kopec 7, 656 53 Brno, Czech Republic

## Abstract

Protein dimerization is a crucial biological process in which proteins interact into homo- or heterodimers to form a functional assembly. Understanding and modulating the molecular mechanisms of protein dimerization and their function represent the cutting edge of research and provide multiple entries for biomedical applications. Label-free methods sensitive to homodimer formation are still required. Electrochemical methods are sensitive to small structural changes due to the presence of a surface and polarization to high negative/positive potentials, where partial denaturation/unfolding can appear. Since the dimeric structure is usually more flexible, the electric field effects induce more salient structural changes close to the electrode surface, accompanied by higher chronopotentiometric/voltammetric responses. Serum albumin and anterior gradient receptor-2 were studied as model homodimeric proteins.

## Graphical abstract

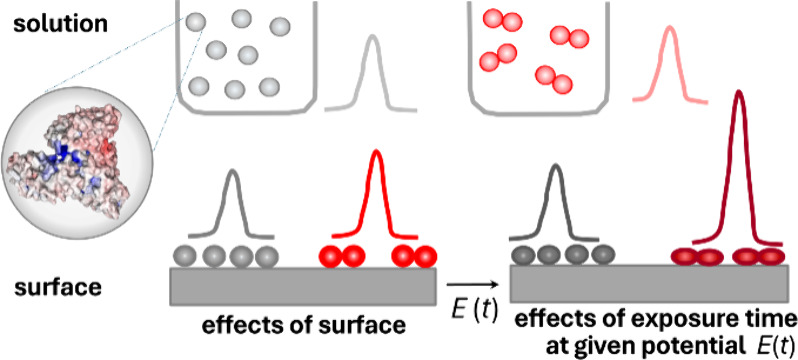

## Introduction

Protein dimerization is a crucial biological process where proteins interact to form homo- or heterodimers, creating functional assemblies. This self-assembly into dimers or higher-order oligomeric aggregates is a common biophysical phenomenon occurring in every cellular compartment. Protein dimerization significantly regulates various cellular pathways, including enzymatic activation, signal transduction, and pathogenic pathways.^1^ Regulating protein dimerization is essential for the growth and development of organisms, influenced by intrinsic or extrinsic factors in the natural environment.^2^ Approximately 50% of oligomeric proteins exist as homodimers.^3^ Dimeric protein forms offer potential advantages, such as genetic savings, functional gains, and structural benefits in comparison to monomeric ones. Notably, several oncogenic signaling pathways are mediated by dimeric proteins, leading to cell proliferation. Many of these hetero- or homodimeric proteins are key components in oncogenic signaling pathways and have become popular targets for developing antitumor agents.^4^ Therefore, understanding and modulating the molecular mechanisms of protein dimerization and their functions could be useful in pharmacology as well as other biomedical applications.

There are many standard methods based on label-free and label-based approaches, which are used for the analysis of oligomers, including dimers. Most of the label-based methods utilize fluorescent tags in various modes of fluorescence analysis, such as steady-state fluorescence, fluorescence anisotropy, Forster Resonance Energy Transfer (FRET), etc.^5–7^ Gel electrophoresis, size exclusion chromatography, ultracentrifugation, dynamic light scattering, nuclear magnetic resonance, X-ray crystallography, microscopy, protein charge transfer spectra, and others are often used label-free methods^8–10^ for this purpose. Some of them are able to distinguish monomers and dimers based on size, and others describe protein structure in detail. Computer simulations offer extraordinary insights into interaction mechanisms. It can reflect binding conformation, interaction forces, binding affinity, key residues, and other information that physicochemical experiments cannot reveal in a fast and detailed manner.^11^ However, the results of simulations must be rigorously tested by using physicochemical experiments. Electrochemical methods can be used for this purpose as techniques for conducting fast preliminary tests of protein structural changes and for elucidating protein interactions with interacting partners, including DNA, proteins, and peptides.^12,13^ Valuable approaches for analyzing biological molecules at a charged surface include voltammetric and impedance spectroscopic methods.^14^ Constant current chronopotentiometric stripping (CPS) is another useful electrochemical method, enabling the acquisition of structural and stability data and providing additional insights into the differential dynamic behavior of the proteins. Like other electrochemical methods, it is characterized by low equipment costs, low price, and relatively simple operation.^12^ The CPS in combination with a catalytic hydrogen evolution reaction allows the study of any known proteins at charged surfaces. The charged surface plays a crucial role in this type of analysis since the proteins are accumulated at the uncharged surface^15^ where they retain their folded structures. Subsequently, electrode polarization to negative potential can lead to structural change in an extreme case, to denaturation/unfolding.^16^ Protein structural changes occur on a wide range of time scales, from the extremely fast (femtoseconds) to the relatively slow (microseconds or even milliseconds).^17^ The exposure time in CPS can be limited to milliseconds and controlled by the value of the stripping current.^13^ Small structural changes in proteins can affect their stability at negatively charged interfaces, potentially leading to unfolding. This is reflected in significant changes in CPS peak H height and shape due to different accessibility of electroactive groups.^18^ CPS has been successfully used to monitor various protein structural changes, such as one amino acid exchange,^19^ oligomerization, and aggregation,^12,18^ with results aligning well with other methods like fluorescence spectroscopy, dynamic light scattering, gel electrophoresis, and H/D exchange mass spectrometry.^20–22^ Several studies were done using model protein serum albumin^23–27^ followed by application to biomedically important proteins.^12,13^

Serum albumin, with a molecular weight of 66.5 kDa, the most abundant protein in human blood plasma,^28^ makes up about half of the serum protein at concentrations between 526 and 753 μM.^29^ Besides maintaining plasma oncotic pressure, serum albumin has various functions, such as transporting steroids and binding to reactive oxygen species.^6^ The human serum albumin (HSA) dimers serve as biomarkers for oxidative stress and liver cirrhosis.^30^ Under physiological conditions, albumin can undergo concentration-dependent, reversible self-oligomerization,^10^ accompanied by a conformational change from a heart-shaped tertiary structure to an ellipsoid in solution.^31^

In this work, we compared CPS responses of monomers and dimers/oligomers, and we show that CPS can distinguish between them utilizing the different adsorption of these individual forms on a charged surface. CPS peak height of native untreated bovine serum albumin (BSA) is between the peaks of the monomeric and dimeric forms. Similar results were observed by using voltammetry at carbon electrodes. Besides serum albumin, we were able to follow redox-dependent dimerization of the protein anterior gradient receptor 2 (AGR2).

## Materials and Methods

### Materials

All chemicals and reagents were purchased from Merck (Czech Republic) of the highest available quality, mostly of analytical grade. Solutions were prepared from triple-distilled water.

Bovine serum albumin (BSA, M.W. 66 432 Da) was obtained from Merck (Czech Republic).

AGR2^21–175^ (lacking signal peptide) cloned into pEHISTEV was kindly provided by Prof. T. Hupp.^32^ AGR2 protein was prepared using a QuikChange Site-Directed Mutagenesis Kit (Stratagene) according to the manufacturer’s instructions. Purification of AGR2 protein was described earlier.^21,33^ Purified fusion His_6_-AGR2 protein was subsequently cleaved with the His_6_-TEV protease to remove the His_6_-tag. The N-terminal His_6_-tag of AGR2 with His_6_-TEV protease was then captured using a HisTrap FF 5 mL column (GE Healthcare), whereas the purified recombinant protein was present in the flow-through fractions. After purification, the protein concentration was determined spectrophotometrically by using the molar extinction coefficient obtained from the ProtParam software on the EXPASY server.

### Procedures

#### Denaturation

BSA at 100 μM concentration in triply distilled water was treated at 95 °C for 30 min, followed by cooling to room temperature in a water bath.

#### Monomer vs Dimer/Oligomer Separation Using Filtration

BSA solution was treated through a Microcon 100k membrane filter (Merck, Germany) with a 100 kDa cutoff by 5 min filtering at 13400 rpm to separate monomers and higher oligomers.

#### Treatment with Hydrogen Peroxide

A 20 μM amount of AGR2 was incubated with different H_2_O_2_ concentrations overnight.

### Electrochemical Analysis

#### Chronopotentiometric Stripping

Chronopotentiometric stripping was performed using an Autolab analyzer (PGSTAT302, Metrohm-EcoChemie, The Netherlands) connected with VA-Stand 663 (Metrohm, Switzerland). A three-electrode system in a standard thermostatic cell was used: hanging mercury drop electrode (HMDE, 0.4 mm^2^) as the working electrode, Ag|AgCl|3 M KCl as the reference, and Pt wire as the auxiliary electrode. Data was collected using GPES version 4.9.007. Experiments were replicated at least 3 times for each measurement. The relative standard deviation (RSD) of voltammetric measurements at the HMDE did not exceed 6%. For most experiments, adsorptive stripping transfer was performed: 1 μM BSA in 50 mM Na-phosphate, pH 7.0, was adsorbed at the HMDE from a 5 μL drop at an open current circuit for the accumulation time *t*_A_ of 60 s and transferred to the background electrolyte, 50 mM Na-phosphate, pH 7.0, where the chronopotentiogram was recorded with a stripping current, *I*_str_, of −70 μA after a 5 s exposure at a potential of *E*_B_ of −0.3 V (if not stated otherwise). The initial potential was the same as that of *E*_B_. Measurements were acquired in open air at 25 °C for serum albumin and at 18 °C for AGR2 protein.

#### Square Wave Voltammetry

An edge plane pyrolytic graphite electrode (ePGE, 4 mm^2^) or a glassy carbon electrode (GCE, 3.14 mm^2^) was used as the working electrode. The samples were adsorbed at the carbon electrode from 8 μL drops at an open circuit potential for a *t*_A_ of 5 min. The electrode was washed with water and transferred to the background electrolyte in the electrochemical cell. The voltammograms were measured from 0.4 to 1.3 V at an amplitude of 10 mV, a step of 10 mV, and a frequency of 26 Hz under argon.

AGR2 experiments were performed in 0.1 M Na-phosphate, pH 7.0, using GCE at 18 °C, while those with BSA were performed in 50 mM Na-phosphate, pH 7.0, at ePGE and 25 °C.

### Differential Scanning Calorimetry

Thermograms of 47 μM BSA denaturation in 50 mM Na-phosphate, pH 7.0, were recorded using a NanoDSC differential scanning calorimeter (TA Instruments, New Castle, USA). The degassed protein solution was heated from 293 to 363 K at a constant rate of 1 K·min^–1^ in a 300 μL capillary cell of the calorimeter. The reference cell was filled with 50 mM Na-phosphate, pH 7.0.

### Electrophoretic Mobility Shift Assay

Native PAGE was performed on Novex WedgeWell Tris-Glycine Mini Protein Gel, 4–20% (ThermoFisher Scientific). Five μg of BSA was loaded on the gel, and PAGE was run in 25 mM Tris and 192 mM glycine running buffer at 225 V for 1 h. The gel was stained in a Coomassie Brilliant Blue solution.

### Fluorescence Spectroscopy

Fluorescence spectra of 2 and 20 μM BSA in 50 mM Na-phosphate, pH 7.0, were measured at an excitation wavelength of 280 nm with an ISS PC1 photon counting spectrofluorometer (ISS, USA) in a quartz cuvette with a 1 cm path length. The excitation and emission slit widths were fixed at 4 nm each.

### Circular Dichroism Spectrometry

Circular dichroism spectra of 2 μM BSA in 10 mM Na-phosphate, pH 7.0, were measured from 180 to 300 nm at 100 nm·min^–1^ scan speed in a quartz cuvette with a 1 mm path length under nitrogen as the average of four accumulations. A J-815 dichrograph (Jasco, Japan) was used for the measurements.

### Electron Microscopy

Monomeric, dimeric, and oligomeric BSA were studied using an environmental scanning electron microscope (ESEM) Quanta 650 FEG, which is well-suited for the observation of a wide range of samples.^34,35^ Presented images were obtained in dark field (DF) mode with a detector for scanning transmission electron microscopy (STEM). Observation was realized in high vacuum mode of the ESEM^36^ using electron beam energy of 30 keV and current of 20 pA. Samples were prepared by application of 1 μL of 15 μM BSA solutions on a TEM grid covered with a holey carbon film and air-dried.

## Results and Discussion

### Chronopotentiometric Stripping of Monomeric and Dimeric/Oligomeric Serum Albumin

We analyzed BSA monomers and dimers using CPS and other methods. Monomers and dimers/oligomers were prepared by filtration through a Microcon 100 kDa. The monomers with a MW of 66 kDa passed through the filter, while dimers and higher oligomers stayed at the upper part of the filter. Such prepared samples were characterized by the native mobility shift assay and used for further analyses. Monomers that passed through the filter yielded a stronger band at MW 66 kDa (Figure 1A, inset), and dimers/oligomers yielded bands corresponding to different quaternary structures (monomers, dimers, and higher oligomers in lower amounts). The presence of monomers could be due to the disaggregation of noncovalent dimers/oligomers by the electric field effects during electrophoresis.^37^

**Figure 1:**
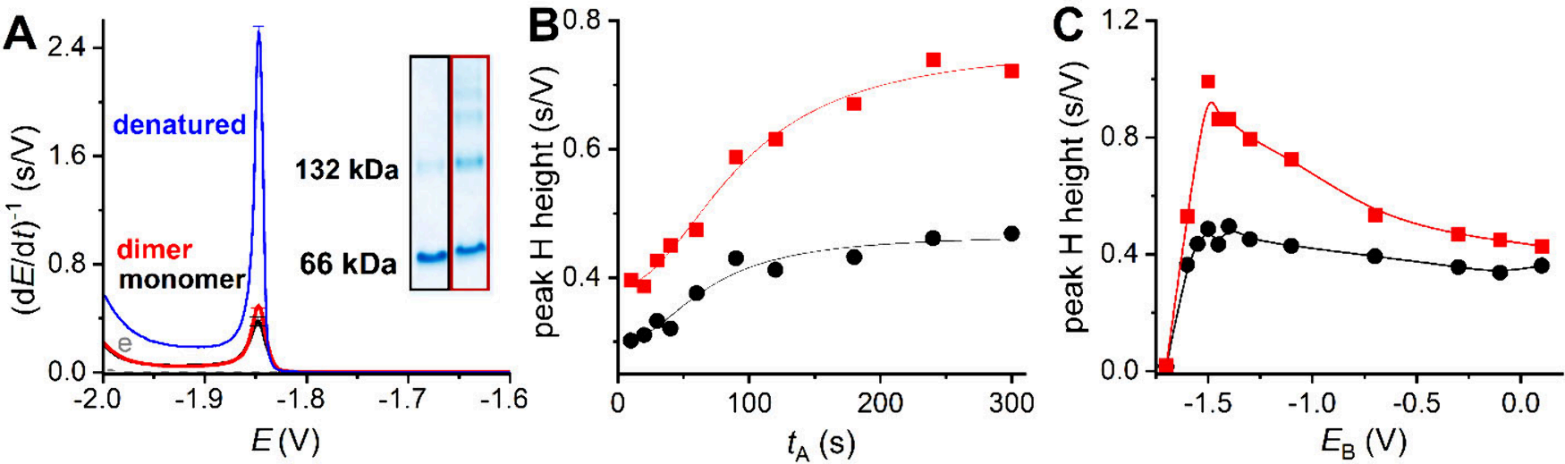
(A) CPS peaks H of 1 μM monomeric (black), dimeric/oligomeric (red), and thermally denatured BSA (blue) in background electrolyte, e. Inset: Native PAGE of BSA was performed for two fractions after filtration through a Microcon. (B,C) Dependence of CPS peak height of monomeric (black) and dimeric/oligomeric BSA (red) on (B) accumulation time, *t*_A_, and (C) potential, *E*_B_, applied before chronopotentiogram recording for 5 s. *I*_str_ = −70 μA.

In CPS, 1 μM BSA was adsorbed from a 5 μL drop on HMDE for an accumulation time, *t*_A,_ of 60 s at open circuit. Then, the BSA-modified electrode was exposed to the potential, *E*_B_, usually of −0.3 V for 5 s, accompanied by stirring, followed by CPS peak H recording. Both samples, monomeric and dimeric/oligomeric BSA, produced a small peak H (about 0.4 s/V at *I*_str_ −70 μA) at potential −1.85 V (Figure 1A) in comparison to thermally denatured BSA, reaching 2.5 s/V. The peak H of monomeric BSA was about 30% smaller than that for the dimeric/oligomeric BSA form. According to the literature, dimer formation leads to a more hydrophobic environment of the ligand-binding pocket.^6^ A more hydrophobic environment promotes stronger adsorption at the mercury surface, resulting in a higher peak H. A similar trend was observed for another protein, galectin-1. In our study,^38^ a smaller peak H was recorded for monomeric galectin-1 incubated at 2 μM, whereas a significantly higher peak H was detected for the homodimeric form incubated at 14 μM, suggesting a shift toward dimer formation at higher concentrations. The dimerization constant for galectin-1 was determined to be around 5 μM.^39^ These results support the notion that dimerization enhances galectin-1 interaction with the electrode surface, probably due to increased hydrophobicity combined with reduced structural compactness.

The difference between monomers and dimers/oligomers was observed for various surface concentrations, changing according to the accumulation time, *t*_A_ (Figure 1B). The highest peak H heights for monomers are achieved at shorter *t*_A_ (about 90 s) than for dimers/oligomers (*t*_A_ > 200 s) probably due to the different diffusion coefficients of monomers and dimers/oligomers and/or preferential adsorption of monomers on the electrode surface. Differences in peak H height reflect the accessibility of electroactive species.^12,18^ More amino acid residues, responsible for higher peak H, are accessible in dimers/oligomers than in monomers,^18^ which could be a result of the different way of adsorption. The adsorption of both species could be influenced not only by differences between monomers and dimers/oligomers themselves, but also due to conformational changes of the monomeric subunits in dimers/oligomers in comparison to the monomer structure.^40^

The dependence of the peak H height of both forms on potential *E*_B_ differed (Figure 1C). At positively charged and slightly negatively charged electrode, the peak height ratio of both forms of BSA changed only slightly. The exposure to potential *E*_B_ from −1.0 V to −1.5 V induced a gradual increase in the difference between monomeric and dimeric/oligomeric forms, with the highest difference at −1.5 V, where the dimeric/oligomeric form yielded peak H twice as high as the monomeric form. At highly negative potentials, more than −1.55 V, both samples were desorbed from the surface, accompanied by a peak H decrease. The data of the *E*_B_ dependence show that applying the potential, *E*_B_, at the protein-modified electrode can enhance differences between monomers and dimers/oligomers.

### Stability of Monomers and Dimers

The CPS peak H height of the dimeric/oligomeric form at potential −1.5 V was more than twice as high as at a potential of −0.3 V, while the peak H of the monomeric form increased by less than 40% (Figure 1C), which suggested its higher stability against electric field effects. A similar conclusion can be drawn from the dependence of CPS peak H on stripping current, *I*_str_ (Figure 2A), which shows a shift of *I*_str1/2_ values to higher negative values for the dimeric form.^18^ This shift implies that shorter exposure to negative potential induces structural protein change.^13,16^ Dimeric/oligomeric forms showed lower stability not only against the electric field effect (Figures 1C and 2A), but also against thermal denaturation, as differential scanning calorimetry proved (Figure 2B). 47 μM BSA in 50 mM phosphate buffer, pH 7.0, in a final volume of 300 μL was heated from 20 to 90 °C with a heating rate of 1 °C/min. The dimer/oligomers denaturation begins at a *T*_i_ of about 42.4 °C and reaches the denaturation temperature (*T*_d_) of about 59.2 °C, while for monomer, a *T*_i_ of about 46.4 °C and *T*_d_ of 59.9 °C were observed, in agreement with data.^41^ The enthalpy of denaturation, Δ*H*, was about 517.5 kJ mol^–1^ for dimers/oligomers and about 687.5 kJ mol^–1^ for monomeric BSA, showing the higher thermal stability of monomers. The fluorescence spectra at the two concentrations differed only in the range of experimental errors (Figure 2C,D). One concentration was chosen, similar to that at which electrochemical experiments were performed, and the second was ten times higher. We tested two concentrations because research by Bhattacharya et al. with native BSA and HSA shows their different behavior at lower and higher concentrations than 10 μM.^9^ The authors explained this as a result of the dimerization. However, we also observed several times higher fluorescence spectra for 20 μM BSA than for 2 μM, but no difference between monomers and dimers/oligomers (Figure 2C,D). Spectra at both concentrations suggest the same environment of tyrosine and tryptophan residues in monomeric and dimeric/oligomeric forms in solutions. Likewise, the differences observed using circular dichroism were negligible (Figure 2E), suggesting only minor structural changes in the albumin structure of monomers and dimers/oligomers. The authors in ref 9 assumed the formation dimer by changing albumin concentrations. The changes in fluorescence and circular dichroism spectra obtained in the mentioned publication are probably due to phenomena other than dimerization.

**Figure 2:**
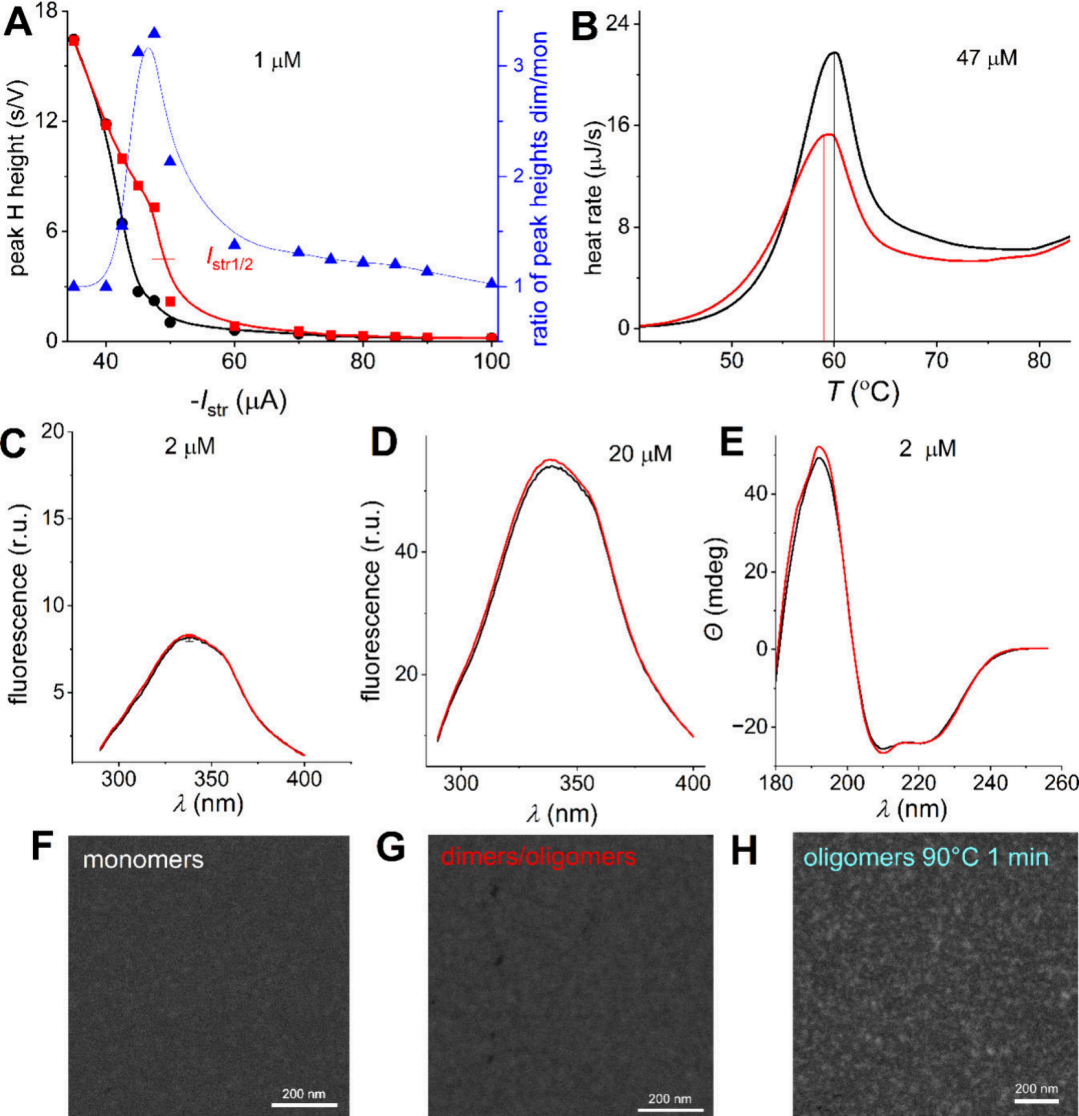
Monomers’ (black) and dimers/oligomers’ (red) stability. (A) Dependence of CPS peak height on stripping current for 1 μM BSA forms. (B) Differential scanning calorimetry of 47 μM BSA monomers and dimers/oligomers. (C, D) Fluorescence spectra and (E) circular dichroism of (C, E) 2 μM and (D) 20 μM BSA forms. (F–H) STEM images of (F) monomeric, (G) dimeric/oligomeric, and (H) higher oligomeric BSA layer. Higher MW oligomers were prepared by treatment at 90 °C for 1 min.

Insignificant changes between layers of monomers and dimers were also proved by STEM visualization in the dark-field mode.^18^ Monomeric (Figure 2F) and dimeric BSA (Figure 2G), as small bright spots (approximately up to 50 nm in diameter), are evenly distributed. Bigger amounts and more intense bright smears were observed for oligomers received after 1 min of incubation at 90 °C (Figure 2H).

Significant differences in electrochemical responses and negligible ones in fluorescent spectra, circular dichroism, and STEM images could be due to the presence of a charged surface. The surface itself may enhance differences between monomers and dimers and oligomers by their different adsorption. Surface polarization can further increase the differences in CPS responses of monomers and dimers/oligomers due to their various destabilization by electric field effects. The electric field effects are a result of the combination of the applied potential and exposure time. A proper combination of applied potential and exposure time is important for the required differences in responses between monomers and dimers/oligomers. For instance, at *I*_str_ value of −100 μA, the peak H heights of both forms are almost the same, since the exposure time is very short (about 1 ms).

### Concentration Dependence

Various sensitivities to changes between monomers and homodimers for different methods could be dependent on the protein concentrations required for the analyses. The results from different methods could be influenced by the protein oligomerization status since concentration is one of its regulatory factors.^10^ The tertiary and quaternary structure of HSA depends on the overall HSA concentration in solution and naturally alters the protein behavior.^6^ We performed CPS analysis of both forms depending on the concentration. Since the CPS peak H height/area depends on concentration, we incubated serum albumin samples at different concentrations overnight, and then the incubated samples were diluted to 1 μM concentration immediately before analysis.

Peak H height of monomeric BSA incubated overnight reached values between 0.35 and 0.64 s/V (Figure 3A), while dimeric BSA reached values between 0.75 and 1.62 s/V (Figure 3B) at *I*_str_ = −60 μA. The average peak H height for untreated BSA at concentrations of 1 and 2 μM was about 0.75 s/V. This value between those for monomers and dimers (Figure 3C) suggests the presence of monomers and dimers/oligomers in the untreated BSA sample. We obtained only an insignificant increase of peak H heights at higher concentrations than 10 μM, which is a concentration that should have caused dimer formation as described Lahiri et al.^6^ However, the equilibrium between monomers and dimers/oligomers can be shifted on the electrode in comparison to solution.^40^ After overnight incubation, the difference between the two forms still remained (Figure 3A,B). This fact contradicts the observation of Bhattacharya et al.^9^ describing fast association and dissociation of monomers to dimers and vice versa depending on concentration. The disagreements between our results and those in ref 9 can be due to more complex processes involved in protein concentration increase, not just simple association with dimers. One can be, for instance, a formation of covalent and noncovalent dimers. Each experimental detail can play an important role.

**Figure 3:**
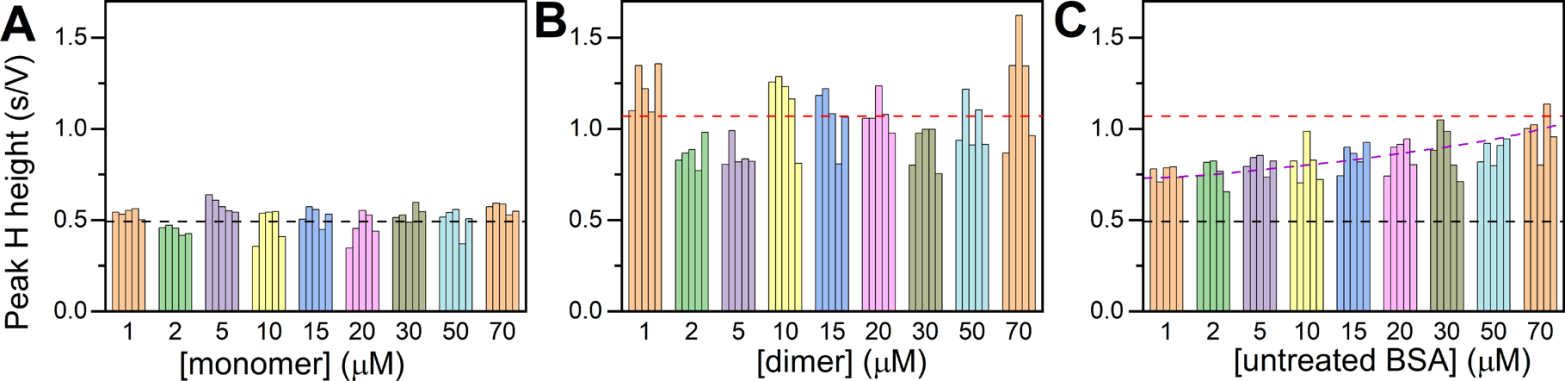
Dependence of peak H height on concentration of (A) monomers, (B) dimers/oligomers, and (C) untreated native BSA at *I*_str_ = −60 μA. The dashed lines represent average values for monomers (black), dimers/oligomers (red), and untreated BSA (violet), where the average value is calculated for each concentration.

### Monomers and Dimers/Oligomers at Carbon Electrodes

We were interested in whether the difference between monomeric and oligomeric forms is due to the charged surface and/or the special properties of the mercury electrode surface. Besides the mercury electrode, the structure-sensitive analysis can be performed at graphite electrodes,^12^ where the best results for native and denatured serum albumin were observed at an edge-oriented pyrolytic graphite electrode and a glassy carbon electrode.^42^ In contrast to the mercury electrode polarized to negative potentials, carbon-based electrodes are polarized to positive potentials.^13,43^ Untreated native, monomeric, and dimeric/oligomeric BSA at 1 μM concentration were adsorbed at ePGE for 5 min, and then the modified electrode was washed and transferred to an electroanalytical cell, where square-wave voltammetric analysis was performed. Likewise, at the HMDE, we observed differences between untreated, monomeric, and dimeric/oligomeric forms, where monomeric and untreated BSA yielded oxidation peaks of tyrosine and tryptophan (peak YW) about 7 times smaller than that for thermally denatured BSA^42^ (Figure 4A).

**Figure 4:**
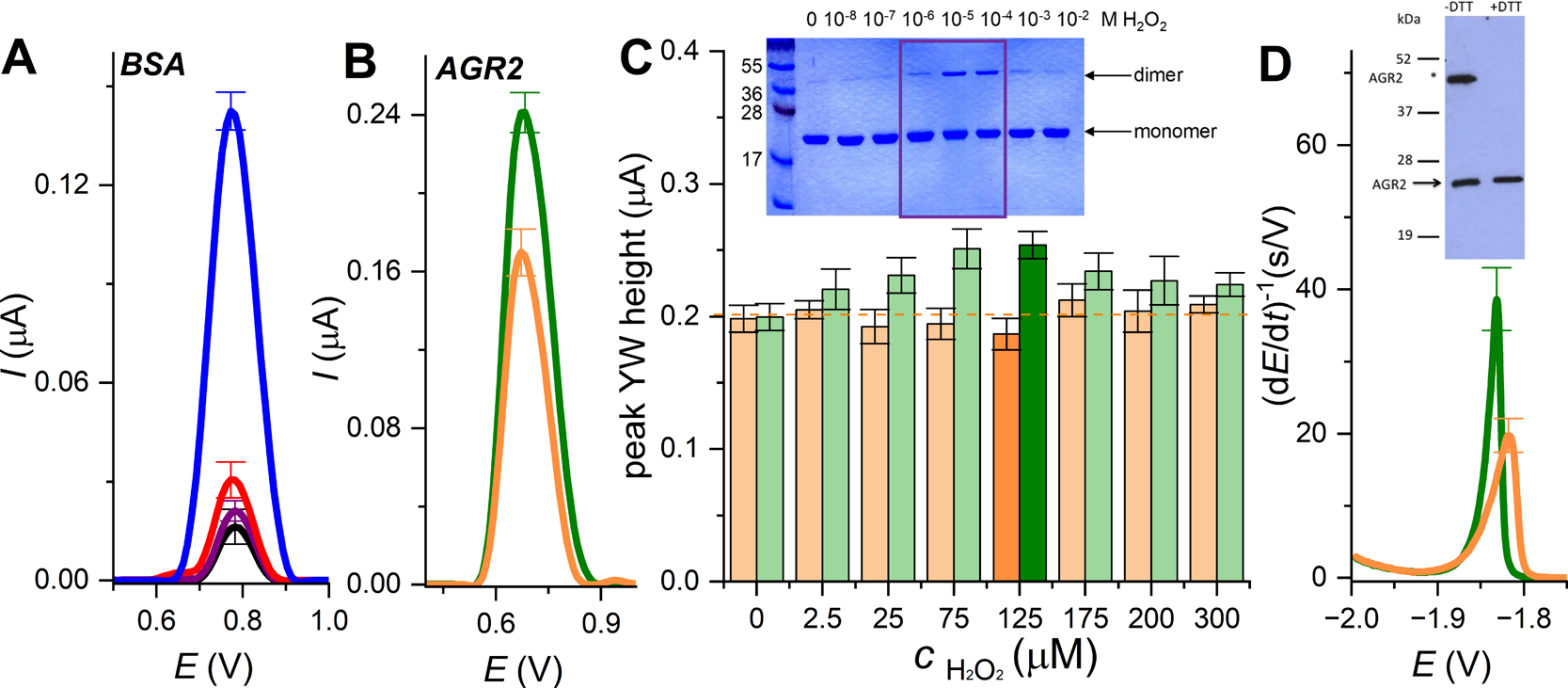
Baseline-corrected square wave voltammetric YW peak at glassy carbon electrode. (A) One μM monomeric (black), dimeric/oligomeric (red), untreated (purple), and denatured BSA (blue) at edge-oriented pyrolytic graphite electrode. (B) Five μM native (orange) and oxidized AGR2 (green) by 125 μM hydrogen peroxide. (C) Dependence of peak YW height on incubation with different concentrations of hydrogen peroxide analyzed in 0.1 M Na-phosphate, pH 7.0. Inset: SDS PAGE of AGR2 incubated with various hydrogen peroxide concentrations. Adapted with permission from ref 47. Copyright 2016 Elsevier. (D) CPS peak H of 2 μM AGR2, *I*_str_ = −15 μA. Inset: SDS PAGE of AGR2 in the absence and presence of reducing agent DTT. Adapted with permission from ref 47. Copyright 2016 Elsevier.

Dimerization of serum albumin in plasma has major implications for normal physiology, since the presence of albumin dimers reduces the osmotic pressure.^44^ Apart from serum albumin, plenty of proteins are active in their homodimeric form. We tested anterior gradient protein 2 (AGR2), which was well characterized by CPS analysis alone,^21,33^ and also in heterodimeric complexes.^45^ This protein is an important dimeric pro-oncogenic protein involved also in respiratory and digestive diseases.^46^ Clarke et al. proved using biochemical assays and electrospray ionization mass spectrometry^47^ that low levels of a chemical oxidant promote an intermolecular disulfide bond through the formation of a labile sulfenic acid intermediate. However, higher levels of the oxidant promote sulfinic or sulfonic acid formation, thus preventing covalent dimerization of AGR2. The redox-dependent monomeric-dimeric formation has implications for the redox regulation of the pro-oncogenic functions of AGR2 protein in cancer cells.^48,49^ We tested whether electrochemical analysis is sensitive to redox-dependent dimerization of the AGR2 protein (Figure 4B–D). Wild type (wt) AGR2 was incubated overnight with different concentrations of hydrogen peroxide, followed by voltametric analysis at a glassy carbon electrode. A significantly higher peak YW was observed for AGR2 treated with H_2_O_2_, between 25 and 175 μM (Figure 4C), which is in good agreement with data obtained using the PAGE mobility shift assay published by Clarke et al.^47^ Additionally, the CPS peak H height of native AGR2 and that oxidized by 125 μM H_2_O_2_ correspond well to the monomeric and dimeric forms under the redox conditions (Figure 4D).

## Conclusion

Protein dimerization is important in many processes in the cell and represents a key step in the respective signaling cascade. Dimers as functional assemblies are less rigid than monomers, although the changes in functionality do not require large conformational changes.^3^ In this work we show that electrochemical methods can monitor processes linked to dimerization. The structure of the monomer and its analogue in dimers differs only negligibly; therefore, studying differences between them is not trivial. Most of the label-free methods that study proteins in solution do not see the difference between them, only those that analyze the difference in MW and radius, such as electrophoretic mobility shift assay, dynamic light scattering, etc. However, these methods are not sensitive to small changes in the structure and functionality.

The advantage of electrochemical methods in protein analysis is that proteins are studied at the surfaces, which are polarized. Protein adsorption on the surface can result in the partial unfolding of the part close to the electrode. Even small conformational changes can influence protein adsorption, in our case, serum albumin, at the electrode surface. Additionally, the electrode polarization to high negative/positive potentials leads to further partial unfolding of the BSA structure in parts close to the electrode. Partial denaturation of the less rigid dimeric structure resulted in a higher chronopotentiometric peak H as well as a higher voltametric peak YW. Besides BSA, a similar trend was observed for native AGR2 and its oxidized form. Thus, our results show the possibility of utilizing electrochemical analysis of homodimeric proteins, which can have a significant impact on understanding biochemical processes, such as in the case of galectin-1.^38^

## Abbreviations

AGR2: anterior gradient receptor 2
BSA: bovine serum albumin
CPS: constant current chronopotentiometric stripping
DTT: dithiothreitol
E_B_: applied potential
ePGE: edge plane pyrolytic graphite electrode
GCE: glassy carbon electrode
HMDE: hanging mercury drop electrode
HSA: human serum albumin
*I*_str_: stripping current
*t*_A_: accumulation time
YW peak: oxidation peak of tyrosine and tryptophan.
